# Cerebral Amyloid Angiopathy Related Inflammation With Prominent Meningeal Involvement. A Report of 2 Cases

**DOI:** 10.3389/fneur.2019.00984

**Published:** 2019-09-23

**Authors:** Agnès Aghetti, Damien Sène, Marc Polivka, Natalia Shor, Sarah Lechtman, Hugues Chabriat, Eric Jouvent, Stéphanie Guey

**Affiliations:** ^1^APHP, Lariboisière Hospital, Department of Neurology and DHU NeuroVasc Sorbonne Paris Cité, Paris, France; ^2^INSERM UMR-S 1141, Paris, France; ^3^Department of Internal Medicine, Lariboisière Hospital, AP-HP, Paris, France; ^4^Univ Paris Diderot, Sorbonne Paris Cité, Paris, France; ^5^Department of Pathology, Lariboisière Hospital, AP-HP, Paris, France; ^6^Department of Radiology, La Pitié-Salpétrière Hospital, AP-HP, Paris, France

**Keywords:** cerebral amyloid angiopathy (CAA), cerebral amyloid angiopathy-related inflammation (CAA-RI), xanthochromia, subarachnoid hemorrhage, meningeal inflammation

## Abstract

Cerebral amyloid angiopathy related inflammation (CAA-RI) is a rare form of CAA characterized by subacute encephalitic symptoms (cognitive decline, seizures, focal deficits) associated with extensive and confluent white matter lesions co-localizing with lobar microbleeds on brain MRI. We report two cases of unusual CAA-RI mimicking meningoencephalitis but without typical brain lesions on FLAIR and T2^*^ sequences. These 2 cases may extend the clinical spectrum of CAA-RI by suggesting the possible occurrence of quite purely meningeal forms of CAA-RI.

## Background

Cerebral amyloid angiopathy related inflammation (CAA-RI) is a very rare disorder resulting from vascular and/or perivascular inflammation in the close vicinity of Aβ deposits. The clinical presentation of CAA-RI is characterized by acute to subacute encephalitic symptoms (confusion, rapidly cognitive decline, focal neurological deficits, seizures, and/or headaches) (1). The MRI pattern is stereotyped with extensive, confluent, more or less symmetric white matter lesions that most often co-localize with cortical microbleeds. A leptomeningeal or parenchymal contrast enhancement may occasionally occur (2, 3).

We report two cases of CAA-RI with prominent meningeal involvement and no parenchymal lesion on brain MRI, whose diagnosis was long delayed.

## Cases Description

Patient 1 was a 60-year old man, without any past medical history, admitted in the emergency room of a nearby hospital in September 2012 for progressively increasing headaches, fever and confusion. The CT scan revealed no parenchymal abnormality. The cerebrospinal fluid examination showed 400 white blood cells/mm^3^ (95% lymphocytes), with high protein amount (2.4 g/L), normoglycorachia, and xanthochromia.

Patient 2 was a 77-yo women with a past medical history of Grave's disease and pulmonary tuberculosis who was admitted in the emergency room in our hospital in January 2018 with a strikingly similar clinical presentation. CT scans showed no parenchymal abnormality. A lumbar puncture revealed 84 white blood cells (90% lymphocytes) with hyperproteinorachia (3.5 g/L), normoglycorachia, and xanthochromia.

Infectious meningoencephalitis was considered in both cases, and probabilistic antibiotic (Penicillin in patient 1, Penicillin, and Cephalosporin in patient 2) and antiviral (Acyclovir for both patients) treatments were started. Despite full dose treatment, cognitive alterations worsened in both patients, with the occurrence of major psychomotor slowing in patient 1 and of aphasia in patient 2.

**Figure 1:**
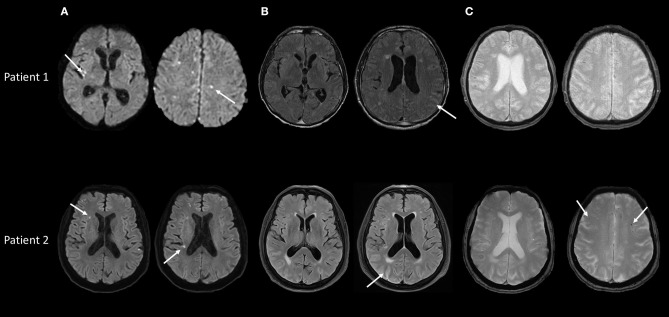
Baseline brain MRI in patients 1 and 2, performed during the first month of the disease. **(A)** Diffusion weighted sequences showing multiple deep and cortical acute microinfarcts in both patients (arrows). **(B)** FLAIR sequences showing no or minimal brain parenchymal lesions but high intensity signal in cortical sulci in patients 1 and 2 (arrows). **(C)** T2* sequences showing doubtful sulcal hypointensities in patient 1 and 5 microbleeds in patient 2 (arrows).

Brain MRI was performed, respectively, at day 15 and day 12 after the first symptoms. None of the two patients had large white matter lesions on the FLAIR sequence. Conversely, high intensity signals were observed along cortical sulci in both cases. On T2^*^ sequences, no microbleeds was detected in patient 1, 5 microbleeds were observed in patient 2. By contrast, multiple cortical and subcortical punctuate lesions on diffusion weighted imaging (DWI) sequences were present in both patients (Figure 1). There was no parenchymal or meningeal gadolinium enhancement. Angio-MRI was normal in both patients. Conventional angiography (in patient 1) and angio-CTscan (patient 2) did not show any large and medium-sized arteries alterations.

Endocarditis and other sources of cardiac emboli were ruled out with transthoracic and transesophageal echocardiography and blood cultures in both patients. Infections screening was negative both in the blood and in the cerebrospinal fluid. Autoimmune and neoplastic etiologies were ruled out by a complete work up, including antinuclear, anti-neutrophil cytoplasmic, anti-cardiolipid, and onconeuronal antibodies. Thoracoabdominopelvic CTscan was normal in both patients. In addition, Positron Emission Tomography scan with fluorodeoxyglucose, gastric endoscopy, skin and ophthalmologic exams were normal in patient 2. Repeated lumbar punctures showed the persistence of xanthochromia with aseptic pleiocytosis in both cases. The association of diffuse subarachnoid hemorrhage and multiple cortical and subcortical microinfarcts finally led to suspect vasculitis involving the small cerebral and meningeal vessels as the potential underlying cause. A brain biopsy was performed in the two patients.

**Figure 2:**
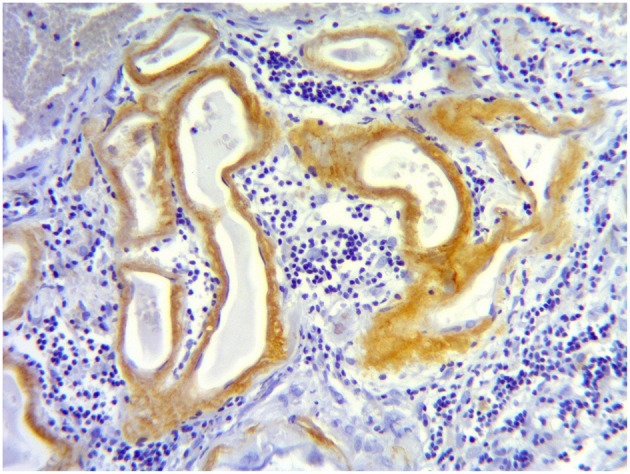
Meningeal biopsy in patient 1. Meningeal tissue section showing Aβ amyloid deposits in the wall of small meningeal arteries (brown staining) and a lymphocytic perivascular infiltrate (dark blue cells).

Pathological examination showed vascular amyloid deposits in leptomeningeal and cortical vessels in both patients. In patient 1, a meningeal perivascular lymphocytic and macrophagic inflammation was observed in the close vicinity of amyloid deposits, leading to the diagnosis of CAA-RI (Figure 2). Cortical vessels were sparred by inflammation. Significant intramural or perivascular inflammation lacked in patient 2, but the tissue was sampled from an area relatively sparred by lesions as seen on MRI. Considering the negativity of the complete work-up, the diagnosis of CAA-RI was considered probable for patient 2.

**Figure 3:**
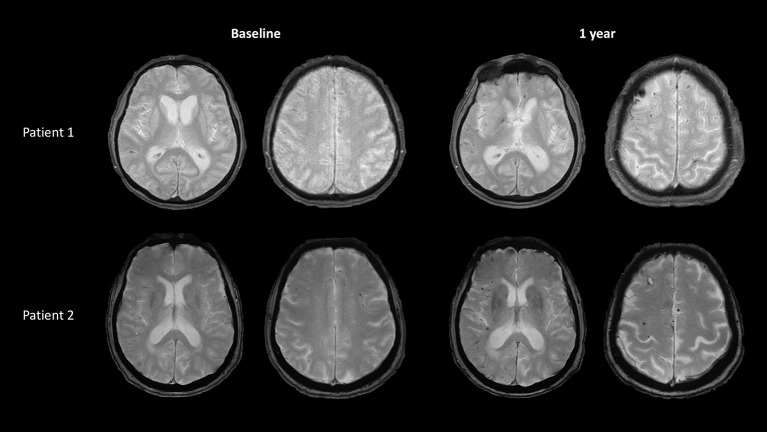
Follow-up brain MRI in the 2 patients. T2* sequences at 1 year after the onset, in patient 1 and patient 2 showing increasing load of superficial microbleeds and hemosiderosis.

Immunosuppressive treatment was started in both patients, with high-dose parenteral corticosteroids (Methylprednisolone cumulative dose of 3,000 and 1,500 mg for patient 1 and 2, respectively) followed by oral corticoids (1 mg/kg of Prednisone), associated with Cyclophosphamide (one infusion per month for 6 consecutive months). No new DWI lesion was noted on control MRI obtained, respectively, at 1 year after the end of treatment for patient 1 and at 1 month in patient 2. In contrast, on T2^*^ sequences, a large number of microbleeds and several foci of cortical siderosis appeared in both cases as previously reported in cerebral amyloid angiopathy (Figure 3). Cerebrospinal fluid examination progressively normalized in both patients. After 2 months, cerebrospinal fluid was acellular and proteinorachia was at 0.5 g/L in patient 1 and 0.3 g/L in patient 2. Patient 1 remained demented with frontal symptoms, apraxia, and disorientation in time and space, whereas patient 2 significantly improved and recovered a full autonomy in daily life. Cognitive assessment obtained at 9 months in patient 2 showed a complete normalization of language but some persisting alterations of executive performances. In addition, Alzheimer's biomarkers were assessed in the cerebrospinal fluid in patient 2 and showed increased tau protein and reduced beta amyloid 1-42.

## Discussion

In the present two cases, the combination of progressive headache associated with fever and confusion, in the total absence of visible parenchymal abnormality on CTscan, but with pleiocytosis in the cerebrospinal fluid led to suspect initially an infectious meningoencephalitis. The clinical worsening under large probabilistic antiviral and antibacterial drugs and negativity of a large work-up led to reconsider the diagnosis in both cases.

Two major features finally contributed to suspect the involvement of small-sized vessels: the presence of multiple microinfarcts on DWI MRI, and the persistence of subarachnoid bleeding over repeated lumbar punctures, while visible vessels were normal on conventional angiography and angioCT, respectively. In both cases, xanthochromia which was present since the first lumbar puncture was overlooked while potentially helpful for diagnosis.

Analysis of brain tissue that showed vascular Aβ amyloid deposits and peri-vascular inflammation surrounding small meningeal arteries finally led to the diagnosis of CAA-RI in patient 1. In patient 2, although inflammation could not be demonstrated, the exclusion of other etiologies, the detection of CAA on brain biopsy, the favorable outcome under immunosuppressive therapy and mostly, the evolution toward typical imaging features of CAA strongly support the diagnosis of CAA-RI. The patchy and segmental distribution of inflammation may also actually cause false negative results on brain biopsy, as previously reported (3, 4).

These two cases show a presentation strikingly different from the usual MRI pattern of CAA-RI including extensive and confluent white matter lesions co-localizing with lobar microbleeds (2, 3). In our 2 cases, hemorrhagic alterations mostly involved meningeal vessels, translating into diffuse subarachnoid hemorrhage, while brain parenchyma was only the site of multiple punctuate foci of ischemia. The predominance of inflammation on meningeal arteries may explain this unusual presentation.

Diagnostic criteria have been recently proposed for CAA-RI to avoid brain biopsy in the most typical cases and allow quickly starting immunosuppressive treatments (2, 3). Our two cases do not satisfy these criteria and suggest that the clinical spectrum of CAA-RI is more variable than previously expected. Therefore, brain biopsy should be performed in such atypical cases.

Immunosuppressive drugs are currently used in CAA-RI such as corticosteroids and cyclophosphamide. Such a therapeutic strategy was followed in our two cases. The use of aggressive immunosuppressive treatment allowed to resolve the acute inflammatory stage of the disease but as expected did not alter the chronic course of CAA. In both patients, cortical microbleeds and siderosis, usual markers of CAA, subsequently accumulated during the follow-up (Figure 3). Paucity of such classical MRI markers at baseline in the two present cases might suggest that these pure meningeal forms of CAA-RI are more likely to occur at early stage of CAA. This point needs to be confirmed by additional reports.

## Ethics Statement

Both patients provided written informed consent for the publication of their clinical data for research via our local written standard protocol.

## Author Contributions

AA and SG: study design, data collection and interpretation, writing the manuscript. EJ and HC: study design, interpretation of data, revision of the manuscript. MP: pathological data analysis, revision of the manuscript. DS, NS, and SL: data collection and interpretation, revision of the manuscript. AA, SG, DS, MP, NS, SL, HC, and EJ approved the final version of the manuscript.

### Conflict of Interest Statement

The authors declare that the research was conducted in the absence of any commercial or financial relationships that could be construed as a potential conflict of interest.
